# The VAD Scheme versus Thalidomide plus VAD for Reduction of Vascular Endothelial Growth Factor in Multiple Myeloma: A Meta-Analysis

**DOI:** 10.1155/2018/3936706

**Published:** 2018-11-11

**Authors:** Gan-Lin He, Duo-Rong Xu, Wai-Yi Zou, Sui-Zhi He, Juan Li

**Affiliations:** ^1^Department of Hematology, The First Affiliated Hospital, Sun Yat-sen University, Guangzhou, China; ^2^Teaching and Research Section of Advanced Mathematics, Xinhua College of Sun Yat-sen University, Guangzhou, China

## Abstract

The VAD (vincristine-doxorubicin-dexamethasone) regimen has been used for decades to treat multiple myeloma (MM). Based on reports that vascular endothelial growth factor- (VEGF-) mediated angiogenesis is critical for MM pathogenesis, the antiangiogenic compound thalidomide has been added to VAD (T-VAD). However, it remains unclear whether T-VAD is more efficacious than VAD for serum VEGF reduction or if the difference influences clinical outcome. Pubmed, Cochrane library, China Biomedical Literature (CBM) database, China National Knowledge Infrastructure (CNKI) database, Vip database, and Wanfang database were searched for relevant studies published up to June 2017. RevMan5.2 was used for methodological quality evaluation and data extraction. Thirteen trials (five randomized, seven nonrandomized, and one historically controlled) involving 815 cases were included. Serum VEGF was significantly higher in MM cases than non-MM controls (MD=353.01, [95%CI 187.52–518.51], P<0.01), and the overall efficacy of T-VAD was higher than that of VAD (RR=1.36, [1.21–1.53], P <0.01). Further, T-VAD reduced VEGF to a greater extent than VAD does ([MD=-49.85, [-66.28− -33.42], P<0.01). The T-VAD regimen also reduced VEGF to a greater extent in newly diagnosed MM patients than it did in recurrent patients ([MD=-120.20, [-164.60–-39.80], P<0.01). There was no significant difference in VEGF between T-VAD patients (2 courses) and nontumor controls (MD=175.94, [-26.08–377.95], P=0.09). Greater serum VEGF reduction may be responsible for the superior efficacy of T-VAD compared to VAD.

## 1. Introduction

Multiple myeloma (MM) is a heterogeneous disease characterized by the clonal proliferation of immunoglobulin-producing plasma cells. It accounts for 1% of all cancers and 10% of all hematologic malignancies [1–4]. About 86000 new cases of myeloma are diagnosed globally each year [5, 6].

Since the 1980s, the VAD (vincristine-doxorubicin-dexamethasone) regimen has been the preferred therapeutic schedule for MM, with curative efficacy ranging from 50% to 70% [7]. Compared to the older MP (melphalan + prednisone) regimen, VAD is faster acting and does not influence the mobilization of stem cells, which is beneficial for subsequent autologous stem cell transplantation and other treatments. An additional advantage of VAD is relatively low cost, so it is widely used for MM treatment in China and other developing countries [8, 9].

Vacca et al. [10] first reported a substantial increase in microvessel density (MVD) among MM patients that was strongly correlated with the proliferation of myeloma cells. Subsequent studies revealed that serum vascular endothelial growth factor (VEGF) is elevated in MM patients and overexpressed in MM cell lines [11–13]. Further, low serum VEGF level is correlated with the curative effect of therapy, while high serum VEGF is a major prognostic factor for poor outcome in MM patients [14]. The occurrence, development, invasion, and metastasis of cancer rely on the generation of new tumor vascular networks [15], strongly suggesting that serum VEGF influences the development and prognosis of MM through bone marrow angiogenesis. Therefore, antiangiogenic agents, especially drugs acting against VEGF, have become a major focus in the development of new MM treatments. A large number of studies show that thalidomide (THD) can inhibit angiogenesis, suppress cytokine signaling, promote myeloma cell apoptosis, and alter the bone marrow microenvironment for sustained stem cell mobilization [16].

There are many reports on the addition of antiangiogenic drugs (including thalidomide) to VAD chemotherapy for MM treatment. Such studies have shown that this T-VAD regimen decreases VEGF and is an effective curative treatment [8, 17–19]. However, no large-scale systematic study has compared the T-VAD and VAD regimens for effects on serum VEGF or assessed the relationship between serum VEGF reduction and clinical outcome. Therefore, we conducted a meta-analysis of studies reporting serum VEGF changes in patients treated by VAD and (or) T-VAD and evaluated the association of serum VEGF changes with clinical outcome. The primary objective of this study is to analyze the evidence for VEGF reduction as a mechanism for T-VAD efficacy based on randomized, nonrandomized, and historical controlled trials.

## 2. Methods

### 2.1. Search Strategy and Selection Criteria

The trials analyzed in this study were identified through an electronic search of the Cochrane library and the PubMed, Wanfang, China Biology Medicine (CBM), and Chinese National Knowledge Infrastructure (CNKI) databases. The search terms were “multiple myeloma”, “thalidomide and VAD”, “chemotherapy”, “thalidomide”, and “vascular endothelial growth factor”. There were no language and date restrictions in the selection of studies. The initial search was performed from January 2000 to July 2017. Our search was based on Preferred Reporting Items for Systematic Reviews and Meta-Analyses (PRISMA) guidelines [20].

The selection criteria for this study were as follows: (1) randomized or nonrandomized controlled trials (RCTs or NRCTs) of patients with multiple myeloma and (2) trials in which patients in the experimental group received the T-VAD regimen while patients in one control group were treated using VAD alone.

### 2.2. Data Extraction and Quality Assessment

Data were extracted independently by two reviewers, and any disagreements were discussed with a third investigator. The following data were collected: the first author's name, year of publication, clinical stage, chemotherapy regimens, number of subjects, patient age, test conditions, and treatment regimen units. Quality of the RCTs was critically appraised using the Cochrane Collaboration tool for assessing risk of bias (RoB) [21]. Studies were assessed based on the Cochrane Handbook by recording bias risks associated with 6 protocol components: random sequence generation, allocation concealment, blinding of participants, blinding of outcome assessment, incomplete outcome data, and selective reporting. Each of the six items was scored as “low risk”, “unclear risk”, or “high risk” [22]. The NRCTs were appraised using a modified RoB form adjusted to fit the nonrandomized study design. A three-point scale together with summarizing arguments for grading (low RoB, high RoB, or unclear) were used to assess each domain.

### 2.3. Curative Effect Evaluation

Primary outcome was serum VEGF level of nontumor populations, MM patients before treatment, and MM patients treated by VAD or T-VAD.

### 2.4. Statistical Analysis

Data were analyzed using Review Manager Version 5.2 provided by the Cochrane Collaboration. A P<0.05 was considered statistically significant. Heterogeneity among the studies was assessed to determine the most suitable model [23]. When heterogeneity existed, a random-effects method was used; otherwise, a fixed-effects method was used. To evaluate whether the results of the studies were homogenous, we performed Inverse Variance in which homogeneity was considered present at I^2^<50% or P>0.1. Weighted mean difference (WMD) was the principal measure of effect and is presented with a 95% confidence interval (CI).

## 3. Results

### 3.1. Search Results

**Figure 1:**
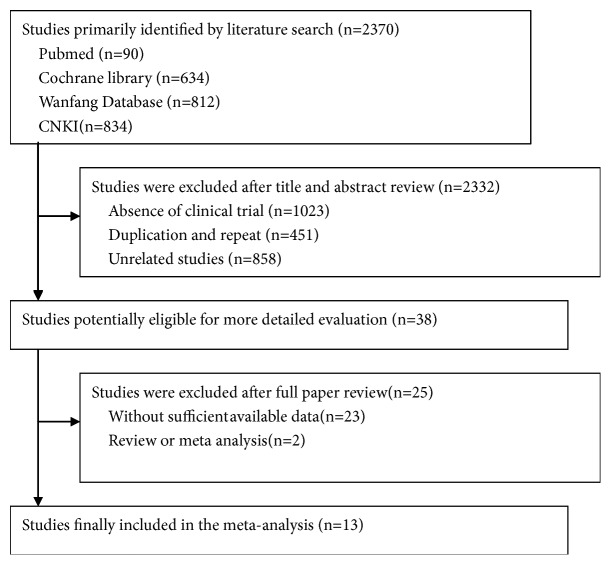
Preferred Reporting Items for Systematic Reviews and Meta-Analyses (PRISMA) flow diagram.

A total of 2370 articles were identified during the initial search. After title and abstract review, 2332 articles were excluded because they were not clinical trials (n=1023), were duplicates (n=451), or did not measure the primary outcome (n=858). In total, 38 studies were selected as potentially relevant. After full-text review, 25 articles were eliminated for insufficient data. Finally, 13 trials with a total of 815 patients were judged eligible for inclusion in this meta-analysis. The reasons for exclusion are illustrated in Figure 1.

### 3.2. Patient Characteristics

The 13 controlled trials included a total of 815 patients [24–36]. Five controlled trials (one randomized and four nonrandomized) including 293 patients were pooled, and data of the 179 MM patients before treatment were compared to the 114 nontumor controls (Table 1). Then, 8 controlled trials (6 randomized, 1 nonrandomized, and 1 historical trial) including 481 MM patients were pooled, and data of 263 patients treated with the T-VAD regimen were compared to 245 patients treated with the VAD regimen (Table 2; 29 patients from one randomized and one nonrandomized trial included in the first comparison of Table 1 were also included in the second comparison of Table 2). Finally, data from 2 randomized controlled trials including 70 VAD-T-treated patients were pooled, and data of 56 patients with initial MM were compared to 14 patients with recurrent MM (Table 3).

### 3.3. Quality Assessment

**Figure 2 fig2:**
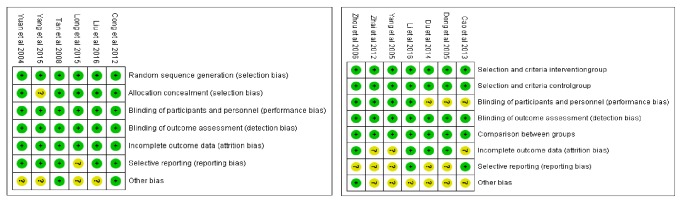
The assessment of the risk of bias.

The risk of bias assessment is shown in Figures 2(a) and 2(b). Overall risk of bias was judged to be low or unclear in the included RCTs. Some of the included studies were described as open-label, or blinding was not reported at all. None of the RCTs fulfilled all six criteria for low RoB; that is, no study described randomization procedures, allocation concealment, or selective reporting with a low RoB (Figure 2(a)). For NRCT and HCTs, the research design was deemed suitable, with low RoB (Figure 2(b)).

### 3.4. Efficacy Assessments

**Figure 3 fig3:**
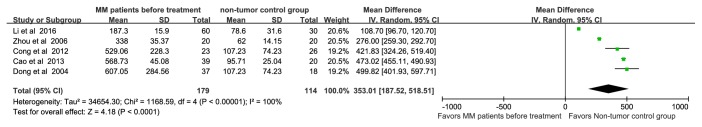
Forest plot comparing serum VEGF between MM patients before treatment and a nontumor control group.

**Figure 4 fig4:**
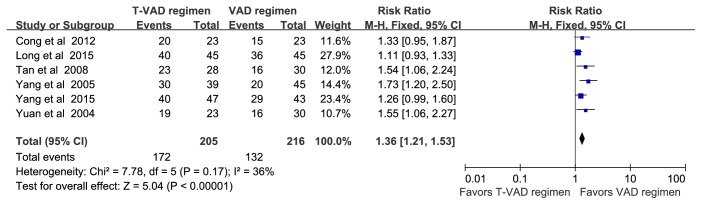
Forest plot comparing overall efficacy of the T-VAD regimen to the VAD regimen for MM treatment.

**Figure 5 fig5:**
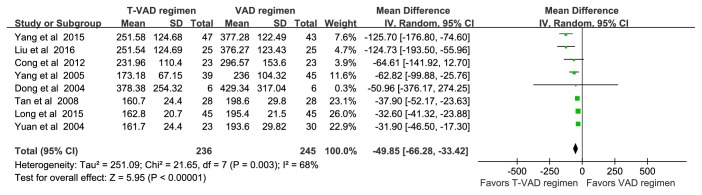
Forest plot comparing serum VEGF levels in MM patients treated with the T-VAD regimen or the VAD regimen.

**Figure 6:**
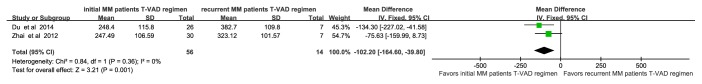
Forest plot comparing serum VEGF between initial and recurrent MM patients treated effectively with the T-VAD regimen [defined as complete remission (CR), very good partial remission (VGPR), or partial remission].

**Figure 7:**
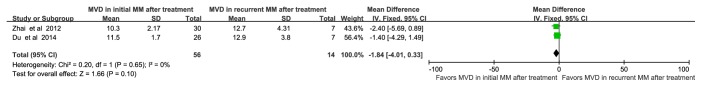
Forest plot comparing marrow microvessel density (MVD) between initial and recurrent MM patients both treated effectively with the T-VAD regimen.

**Figure 8:**
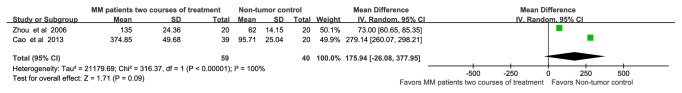
Forest plot comparing serum VEGF levels between MM patients after two courses of T-VAD treatment and nontumor controls.

The forest plot analyses of these comparisons are shown in Figures 1 [Fig fig2] [Fig fig3] [Fig fig4] [Fig fig5]–6. Serum VEGF was significantly higher in MM patients than nontumor controls (MD = 353.01, [187.52–518.51], P<0.01) (Figure 3). Overall efficacy of the T-VAD regimen was higher than that of the VAD regimen (RR = 1.36, [1.21–1.53], P<0.01) (Figure 4). Further, the T-VAD regimen was more effective for decreasing serum VEGF than the VAD regimen (MD=-49.85, [-66.28− -33.42], P<0.01) (Figure 5). The T-VAD regimen was also more effective for reducing serum VEGF in initial MM than recurrent MM, even when treatment was curative in both groups (MD = -120.20, [-164.60–-39.80], P<0.01) (Figure 6). Alternatively, there was no significant difference in marrow microvessel density (MVD) reduction between initial MM and recurrent MM patient groups, both showing curative results (MD = 175.94, [-26.08–377.95], P>0.01) (Figure 7). Finally, two courses of T-VAD reduced serum VEGF to the level of a nontumor control group (MD = 175.94, [-26.08–377.95], P=0.09) (Figure 8).

## 4. Discussion

Vascular endothelial growth factor is a potent inducer of bone marrow angiogenesis, suggesting a significant role in MM pathogenesis. Indeed, high serum VEGF is an independent risk factor for poor MM prognosis, while reduced VEGF is related to the curative effect of treatment [4]. However, there is no large-sample evidence that the antiangiogenic T-VAD regimen is superior to the conventional VAD regimen for reducing serum VEGF or if the difference in VEGF suppression is related to the difference in antitumor efficacy. Therefore, we conducted this meta-analysis of 13 controlled trials. Results indicated that serum VEGF is higher in MM patients than nontumor controls (Figure 3). This suggests that myeloma cells release more VEGF, consistent with previous findings that overexpression of VEGF and aberrant angiogenesis in bone marrow are closely related to MM pathogenesis [41–43]. Thus, new targeted therapies that treat MM by adjusting the bone marrow microenvironment to suppress angiogenesis may be advantageous compared to traditional chemotherapy regimens. Thalidomide was added to the VAD regimen based on its efficacy for antiangiogenesis, for immunosuppression, and for adjusting the bone marrow microenvironment [6, 44, 45], and clinical studies have shown that the overall efficacy of T-VAD for MM is up to 70% [7, 46]. This regimen is also relatively inexpensive and so is particularly valuable for MM treatment in developing countries. This meta-analysis shows that the curative efficacy of T-VAD (76.92% to 88.9%) is greater than VAD (Figure 4) and also more effective than VAD for reducing serum VEGF (Figure 5), suggesting that the enhanced curative efficacy is linked to reduction in serum VEGF.

This meta-analysis also addressed whether the efficacy of T-VAD for reducing serum VEGF is the same for patients with initial and recurrent MM. The decrease in serum VEGF was significantly greater during initial MM than recurrent MM despite curative effects in both groups (Figure 6). Alternatively, there was no difference in MVD reduction (Figure 7) between initial and current MM patients effectively treated with T-VAD. Thus, serum VEGF reduction appears to be a more reliable index of treatment efficacy for initial MM than recurrent MM, although antiangiogenesis appears important for good clinical outcome in both cases. The analysis conducted by the point that the VEGF expression level in bone marrow of multiple myeloma patients is consistent with MVD shows that it is consistent with the viewpoint of referencing it in the treatment of relapsed/refractory MM without standard treatment strategy [47].

Finally, there was no significant difference between serum VEGF in MM patients treated with two courses of T-VAD and healthy controls, underscoring the efficacy of T-VAD for reducing serum VEGF and further implicating elevated serum VEGF in MM pathogenesis and prognosis. However, the P value is relatively small, which may be caused by insufficient sample size, and the possibility that some malignant myeloma cells still remain in the body after effective treatment cannot be excluded. These residual myeloma cells will still release VEGF, and residual VEGF may be one factor promoting recurrence, an issue that requires further study [27].

This meta-analysis has certain limitations. Most of the trials included in the study were conducted in China, which may be related to the low price of this regimen. Additionally, MM mainly afflicts the elderly, so incidence may be related to population aging in China [8]. Moreover, the sample size for the efficacy indicator (serum VEGF) may not be sufficient to detect smaller differences among groups (Figures 4 [Fig fig5]–6). Finally, this analysis did not consider toxic side effects [8, 9]. Given that the side effects of the VAD regimen are significant, it is necessary to develop alternatives.

## 5. Conclusion

This meta-analysis indicates that serum VEGF is a clinically significant factor influencing the occurrence, development, and prognosis of multiple myeloma and that the antiangiogenic T-VAD regimen is a better MM treatment than VAD alone. This superior efficacy is associated with greater suppression of serum VEGF. Thus, serum VEGF is an important indicator of treatment efficacy for first-time MM. Larger scale studies are still required to confirm the superior efficacy of T-VAD and the clinical significance of serum VEGF for MM prognosis and treatment outcome.

## Figures and Tables

**Table 1:** Nontumor patients vs. MM patients before treatment.

Study	Data year	Stage I/II/III	Patients		Age (years) Control/Exp	Comparison		Test conditions and units
			Control (M/F)	Exp (M/F)		Control	Exp	
Cao et al., 2013 [24]	2008−2011	4/8/27^*∗∗*^	20 (9/11)	39 (23/16)	45/59	Healthy control	Before MM treatment	ELISA VEGF (pg/ml)
Li et al., 2016 [25]	2010−2014		30	60 (37/23)	63/67	Non-tumor patient	Before and after treatment	ELISA VEGF (ng/L)
Zhou et al., 2006 [26]	2001	1/ 2/17^*∗∗*^	20	20 (15/5)	48/59	Non-tumor patient	Before and after treatment	ELISA VEGF (ng/L)
Cong et al., 2012 [27]	2008−2011	4/12/30^*∗∗*^	26 (18/8)	23	65/68	Healthy control	Before and after treatment	ELISA VEGF (ng/L)
Dong et al., 2004 [28]	2003	7/ 8/22^∆^	18 (8/10)	37 (29/8)	31.3/52.5	Healthy control	Before MM treatment	ELISA VEGF (ng/L)

*∗*Zhang N, Shen Ti, editor. Blood disease diagnosis and efficacy standards. Version 2. Beijing: Science Press, 1998;373.376 [37]; *∗∗*Zhang Zhinan, Sen Ti. Blood disease diagnosis and efficacy standards. 3rd edition Beijing: Science Press. 2007, 232−235 [38]; Δ Durie BG Salmon SE. A clinical staging system for multiple myeloma. Correlation of measured myeloma cell mass with presenting clinical features response to treatment and survival. Cancer, 1975, 36:842−854 [39]; # Blood Physicians Association, Hematology Branch of Chinese Medical Association, Multiple myeloma working group in China. China guidelines for diagnosis and treatment of multiple myeloma (revised in 2013). Chin J Int Med, 2013, 52(9):791−795 [40].

**Table 2:** VAD-treated vs. T-VAD-treated MM patients.

Study	Stage	Patients		Age (years)	Comparison		Trial conditions and units
	I/II/III	Control (M/F)	Exp (M/F)	Control/Exp	Control	Exp	
Cong et al., 2012 [27]	4/12/30^*∗∗*^	23	23	65/68	VAD	T-VAD	ELISA VEGF (ng/L)
Yang et al., 2015 [29]	6/26/58^*∗∗*^	43 (23/20)	47 (25/22)	67.4/66.9	VAD	T-VAD	ELISA VEGF (pg/ml)
Long et al., 2015 [30]	0/19/71^*∗*^	45 (26/19)	45 (27/18)	56.4/55.3	VAD	T-VAD	ELISA VEGF (ng/L)
Yuan et al., 2004 [31]	10/31/12^*∗*^	30 (18/12)	23 (15/8)	62/61	VAD	T-VAD	ELISA VEGF (ng/L)
Liu et al., 2016 [32]	0/24/26^*∗*^	25 (12/13)	25 (13/12)	67.1/67.1	VAD	T-VAD	ELISA VEGF (pg/ml)
Tan et al., 2008 [33]	11/33/12^*∗*^	28 (16/12)	28 (15/13)	62/61	VAD	T-VAD	ELISA VEGF (ng/L)
Yang et al., 2005 [34]	0/ 8/76^∆^	45 (31/14)	39 (29/10)	51.5/52.3	VAD	T-VAD	ELISA VEGF (ng/L)
Dong, 2004 [28]	7/8/19^#^	6	6	31.3/49.8	VAD	T-VAD	ELISA VEGF (ng/L)

**Table 3:** Initial MM vs. recurrent MM patients treated with T-VAD.

Study	Data year	Tumor stage I/II/III	Patients		Age (year)		Comparison		Test conditions and units
			Initial	Relapse	Initial	Relapse	Initial	Relapse	
Zhai et al., 2012 [35]	2004−2011	0/13/29^*∗*^	30	7	56	56	T-VAD	T-VAD	ELISA VEGF (pg/ml)
Du et al., 2014 [36]	Not clear	0/ 6/32^#^	26	7	58	58	T-VAD	T-VAD	VEGF (pg/ml)

*∗*Zhang N, Shen Ti, editor. Blood disease diagnosis and efficacy standards. Version 2. Beijing: Science Press, 1998;373.376 [37]; *∗∗*Zhang Zhinan, Sen Ti. Blood disease diagnosis and efficacy standards. 3rd edition Beijing: Science Press. 2007, 232−235 [38]; Δ Durie BG Salmon SE. A clinical staging system for multiple myeloma. Correlation of measured myeloma cell mass with presenting clinical features response to treatment and survival. Cancer, 1975, 36:842−854 [39]; # Blood Physicians Association, Hematology Branch of Chinese Medical Association, Multiple myeloma working group in China. China guidelines for diagnosis and treatment of multiple myeloma (revised in 2013). Chin J Int Med, 2013, 52(9):791−795 [40].

## Data Availability

The data supporting this meta-analysis are from previously reported studies, which have been cited. The processed data are available in the forest plot.
